# Community pharmacy interventions for public health priorities: protocol for a systematic review of community pharmacy-delivered smoking, alcohol and weight management interventions

**DOI:** 10.1186/2046-4053-3-93

**Published:** 2014-08-22

**Authors:** Adam Todd, Helen J Moore, Andrew K Husband, Clare Bambra, Adetayo Kasim, Falko F Sniehotta, Liz Steed, Carolyn D Summerbell

**Affiliations:** 1 School for Medicine, Pharmacy and Health, Durham University, Durham TS17 6BH, UK; 2 FUSE, UKCRC Centre for Translational Research in Public Health, Newcastle NE2 4AX, UK; 3 Wolfson Research Institute for Health and Wellbeing, Durham University, Durham TS17 6BH, UK; 4 Department of Geography, Durham University, Durham DH1 3LE, UK; 5 Institute of Health & Society, Newcastle University, Newcastle NE2 4AX, UK; 6 Blizard Institute, Barts and The London School of Medicine and Dentistry, London E1 2AT, UK

**Keywords:** Systematic review, Public health, Interventions, Community pharmacy, Obesity, Weight management, Smoking cessation, Alcohol misuse

## Abstract

**Background:**

Community pharmacists can deliver health care advice at an opportunistic level, related to prescription or non-prescription medicines and as part of focused services designed to reduce specific risks to health. Obesity, smoking and excessive alcohol intake are three of the most significant modifiable risk factors for morbidity and mortality in the UK, and interventions led by community pharmacists, aimed at these three risk factors, have been identified by the government as public health priorities. In 2008, the Department of Health for England stated that ‘a sound evidence base that demonstrates how pharmacy delivers effective, high quality and value for money services is needed’; this systematic review aims to respond to this requirement.

**Methods**/**design:**

We will search the databases MEDLINE, Embase, CINAHL, PsycINFO, Social Sciences Citation Index, ASSIA, IBSS, Sociological Abstracts, Scopus and NHS Economic Evaluation Database for studies that have evaluated interventions based on community pharmacies that aim to target weight management, smoking cessation and alcohol misuse. We will include all randomised controlled trials (RCTs), non-randomised controlled trials (NRCTs), controlled before-after studies (CBAs) and interrupted time series (ITS) and repeated measures studies. Data from included studies will be extracted by two independent reviewers and will include study details methods, results, intervention implementation/costs and methodological quality. Meta-analysis will be conducted if appropriate; if not, the synthesis will be restricted to a narrative overview of individual studies looking at the same question.

**Discussion:**

The review aims to summarise the evidence base on the effectiveness of community pharmacy interventions on health and health behaviours in relation to weight management, smoking cessation and alcohol misuse. It will also explore if, and how, socio-economic status, gender, ethnicity and age moderate the effect of the interventions and will describe how the interventions included in the review have been organised, implemented and delivered, since context is an important factor governing the success of public health interventions. The findings from this review will have an impact on the commissioning of public health services aiming to promote healthy weight, smoking cessation and prevent excessive alcohol consumption.

**Systematic review registration:**

The review has been registered with PROSPERO (registration no. CRD42013005943). Available at: http://www.crd.york.ac.uk/prospero/display_record.asp?ID=CRD42013005943.

## Background

A number of agencies and countries, including the World Health Organization (WHO) and the Department of Health for England, have set a clear agenda for the future of public health. This agenda is focused on improving the healthy life expectancy of the population and, where possible, reducing or removing threats to this aim [1,2]. One strand within this agenda is to create accessible, multi-disciplinary networks of public health professionals who work within communities and provide services to address key public health issues and health inequalities and ultimately improve health and wellbeing.

Worldwide, community pharmacies may be an important component of this agenda; the WHO acknowledges that community pharmacies and their staff are easily accessible and, as such, could play a key role in public health initiatives. Indeed, it is thought that the key characteristic through which public health interventions that are delivered out of community pharmacies may have a positive impact on health equity relates to their access and acceptability. For example, in England, there are over 10,500 community pharmacies, distributed across urban and rural areas [3], allowing the public to access health care without an appointment. These community pharmacies are open at convenient times, including evenings and weekends, allowing access for people who work a wide range of hours. This situation has consistently improved in recent years in England, with policy drives to improve access to medicines, including the promotion of ‘100 hour pharmacies’ , which must open 100 h per week for every week of the year [2]. It has been shown that 89% of the population in England can access a pharmacy from home within a 20-min walk. Importantly, in areas of highest deprivation, this value increases to almost 100% [4].

Estimates vary with regard to the reach of the community pharmacy network, but they tend to be relatively high; a survey published in 2008 found that 95% of the population of Scotland make at least one visit during any 1 year [5]. Thus, in countries such as England, the public has convenient access, at a time of their own choosing, to all services within community pharmacy. In terms of addressing socio-economic inequalities in health, the unique access characteristics of community pharmacies may be more attractive to individuals who cannot, or choose not to, access conventional health care providers. Community pharmacists are also able to deliver health care advice at an opportunistic level, related to prescription or non-prescription medicines and as part of focused services designed to reduce specific risks to health [6]; this may increase the likelihood of such interventions being delivered to those who are ‘hard to reach’.

Although we have postulated above a number of characteristics of the interventions under review which may reduce socio-economic inequalities in smoking, alcohol misuse and obesity, it is worth reflecting on the current literature. It is known that some effective universal public health interventions increase inequalities by disproportionately benefitting less disadvantaged groups (‘intervention-generated inequalities’ or IGIs) [7] and that these can occur throughout an intervention, including at the point of service provision or access, uptake and compliance [8]. Lorenc et al. have conducted a rapid overview of systematic reviews to identify the types of interventions that are more likely to produce IGIs and those that have the potential to decrease inequalities [9].

Obesity, smoking and excessive alcohol intake are three of the most significant modifiable risk factors for morbidity and mortality in middle- and high-income countries [10,11], and interventions which aim to reduce obesity, smoking rates and alcohol misuse, led by community pharmacists and other service providers, have been identified by the Department of Health for England as public health priorities [12,13].

Conditions which are caused or exacerbated by these risk factors include cardiovascular disease, liver disease and certain cancers; socio-economic inequalities in the prevalence and treatment of these conditions are major contributors to overall inequalities in health and wellbeing.

The prevalence of obesity in both children and adults remains relatively high in the UK compared with most other European countries [14,15], particularly in areas of social deprivation. Obesity is known to be a risk factor for coronary heart disease, type 2 diabetes and some cancers, and it is also associated with various other health issues, such as muscular-skeletal and psychosocial problems [16,17]. The prevalence of obesity in women living in the UK is highest amongst those living in areas of social deprivation, but the association in men is less clear [18].

Smoking is associated with the highest number of preventable deaths in the UK [19] with, on average, half of all lifelong smokers dying prematurely, losing on average about 10 years of life [20]. It is estimated that up to 86,500 preventable deaths each year can be attributed to smoking in the UK [21]. Up until 2007, the rates of smoking declined in the UK population to around 21%, but since then this figure has plateaued [22]. In the UK, the rates of smoking are greatest in low socio-economic groups [23].

The number of alcohol-related deaths in the UK is increasing and, since 1991, has almost doubled. As with smoking and obesity rates, higher rates of excessive alcohol intake and alcohol-related deaths are reported in those living in areas of social deprivation [24]. In addition, for men in unskilled low-paid occupations, the rate of alcohol-related mortality is around 3.5 times greater than those in managerial and professional occupations [25]. For women, this figure is even higher, with those in unskilled low-paid occupations at around 5.7 times greater risk of alcohol-related mortality than those in managerial and professional occupations [25].

In almost all regions of the UK, community pharmacies are often the most accessible and available health care provider to the community, and higher numbers of community pharmacies are found in areas of high social deprivation. Because of this, community pharmacies have been identified as potentially ideal settings to deliver public health interventions [2]. As such, many community pharmacies now offer, through locally commissioned services, alcohol, smoking cessation and weight management schemes [26]. These services are delivered by pharmacists, pharmacy technicians and counter assistants with a view to modifying health-related behaviours. Typically, they are not only provided face-to-face in the community pharmacy but can also be delivered via other interactions, such as the telephone or Internet, although this is more common for follow-up appointments. The specific types of interventions are wide ranging and include two main approaches: pharmaceutical-related (e.g. supplying nicotine replacement therapy for smoking cessation) and non-pharmaceutical-related (e.g. providing advice on behaviour change techniques), or a combination of both approaches. At present, in England, many of these services are commissioned by the local authority according to local need; all services are delivered to an agreed framework specification that allows for variations in the delivery of the service at a local level.

Existing reviews relevant to community pharmacy weight management, smoking and alcohol interventions were not able to make a judgement about the efficacy of these interventions [27-29], primarily because the evidence base they included in their reviews was limited or of poor methodological quality, or both. However, trials of good methodological quality have been published since these reviews were published, and others are ongoing or about to start. In 2008, the Department of Health stated that ‘a sound evidence base that demonstrates how pharmacy delivers effective, high quality and value for money services is needed’ , and this systematic review aims to respond to this requirement [2]. We hope that the findings of our review will be of relevance to those responsible for policy and practice in many countries that are trying to tackle obesity, smoking and alcohol misuse, where one option is to deliver interventions through community pharmacies.

### Objectives

The objectives are as follows:

1. To assess the effects of community pharmacy interventions on health and health behaviours in relation to weight management, smoking cessation and alcohol misuse.

2. To explore if (and how) socio-economic status, gender, ethnicity and age moderate the effect of the interventions.

3. To describe how the interventions included in the review have been organised, implemented and delivered, since context is an important factor governing the success of public health interventions.

## Methods/design

We will undertake a systematic review of effectiveness on health and health behaviours in relation to weight management, smoking cessation and alcohol misuse and will carry out the review using the principles outlined in the Cochrane Handbook for Systematic Reviews of Interventions [30]. Prior to conducting the review, we carried out a scoping search as part of the funding application process, which suggested that we would find very few relevant randomised controlled trials (RCTs). We will include evidence from a variety of study designs: RCTs, non-randomised controlled trials (NRCTs), controlled before-after studies (CBAs), interrupted time series (ITS) and repeated measures studies (using Cochrane Effective Practice and Organisation of Care (EPOC) study design criteria [31]) that include a measure of change as an outcome of interest. Within the study designs eligible for this review, we will include all types of studies, including cluster RCTs, and all types of NRCTs.

The review is registered with PROSPERO (registration no. CRD42013005943) and will be reported to the PRISMA [32,33] and TIDieR [34] recommendations.

### Population

The review will include any study that aims to evaluate interventions targeting weight management, smoking cessation and alcohol misuse based in any community pharmacy. Therefore, the studied population will include people of all ages, gender, socio-economic status and nationality.

### Interventions

The review will examine studies that have explored the effectiveness of any intervention that aims to target weight management, smoking cessation or the misuse/excessive consumption of alcohol which were based in any community pharmacy, in any country. Interventions that are led by a pharmacist or the wider pharmacy team but take place outside of the community pharmacy will be excluded.

The rationale behind the important element of the interventions included in this review relates to access and acceptability of the interventions, particularly for those who live in geographical areas of high deprivation and/or are members of sub-groups within the community who are less likely to access health services. Although the goal of the interventions included in this review was to either stop smoking, reduce alcohol intake or reduce body weight, we did not put any restrictions on the type of intervention, for example, in terms of an underpinning theory, methods and materials used and training provision.

### Comparator

Studies with and without comparators will be included in the review. There will be no restrictions on the type of comparator used in the study.

### Outcomes

Studies will be included if they have at least one primary outcome of interest reported.

The review will utilise the causal modelling framework proposed by Hardeman et al. [35] to conceptualise primary and secondary outcomes of behavioural interventions (Table 1). We will only include studies on smoking cessation and alcohol intake if they include a measurement of relevant behaviour as an outcome. For weight loss interventions, we will only include studies if they include a measurement of body weight or fatness (e.g. BMI, body fat) as an outcome. There will be no other restrictions on study inclusions by outcomes reported. To explore causal mechanisms, we will analyse secondary outcome data, where this data is available, in accordance with the causal modelling framework proposed by Hardeman et al. [35]. This framework contains four categories: determinants of behaviour, behavioural outcomes, physiology and biochemical outcomes and health outcomes, as shown in Table 1.

**Table 1:** **Four categories of behaviour outcomes as proposed by Hardeman et al.**[35]

**Intervention**	**The four categories of behaviour outcomes (with examples)**			
	**Determinant of behaviour**	**Behaviour outcomes**	**Physiology and biochemical outcomes**	**Health outcomes**
Community pharmacy smoking cessation	1. Knowledge	Cessation of smoking	Improvements in lung capacity	Incidence rates for lung cancer
	2. Skills			General physical or mental health (e.g. SF8, GHQ-12)
	3. Social role and identity			
	4. Beliefs about capability			Health-related quality of life (e.g. EQ5D)
	5. Beliefs about consequences			
	6. Motivation			Health service use (e.g. GP visits)
	7. Cognitive and decision processes			
	8. Environment			
	9. Social influences			
	10. Emotion			
	11. Behavioural regulation			
Community pharmacy alcohol screening and intervention	1. Knowledge	Reduced score on the Fast Alcohol Screening Test (FAST) demonstrating a reduction in alcohol intake	Improved liver function, reduced drinking, fewer alcohol-related sequelae	Incidence rates for liver disease
	2. Skills			General physical or mental health (e.g. SF8, GHQ-12)
	3. Social role and identity			
	4. Beliefs about capability			Health-related quality of life (e.g. EQ5D)
	5. Beliefs about consequences	Change in alcohol intake		
	6. Motivation	Change in occasions of binge drinking?		Health service use (e.g. GP visits)
	7. Cognitive and decision processes			
	8. Environment			
	9. Social influences			
	10. Emotion			
	11. Behavioural regulation			
Community pharmacy weight management	1. Knowledge	Physical activity	Weight loss, reduction of BMI, reduction in waist circumference	Cardiovascular risk score
	2. Skills	Dietary intake		Incidence rates for CVD
	3. Social role and identity			General physical or mental health (e.g. SF8, GHQ-12)
	4. Beliefs about capability			Health-related quality of life (e.g. EQ5D)
	5. Beliefs about consequences			Health service use (e.g. GP visits)
	6. Motivation			
	7. Cognitive and decision processes			
	8. Environment			
	9. Social influences			
	10. Emotion			
	11. Behavioural regulation			

Classifying behavioural outcomes might be further complicated by studies focusing or incorporating effectiveness of implementation [36]. For example, we might have a range of trials where pharmacies are randomised to conditions in which the pharmacy staff are trained to deliver the intervention.

### Study designs

A rigorous and inclusive international literature search will be conducted for all RCTs and NRCTs. We will also include controlled CBAs and ITS and repeated measures studies (using Cochrane EPOC study design criteria, as described previously). All studies of these design types will be eligible for inclusion, provided that they meet the further inclusion criteria discussed in the ‘Outcomes’ section.

### Literature searching

We will run one overarching search (amended where necessary to suit syntax requirements) to identify studies of relevance for all three topics, and we will include the following electronic database searches (host sites given in parentheses): MEDLINE (Ovid), Embase (Ovid), CINAHL (NHS Evidence Health Information Resources), PsycINFO (NHS Evidence Health Information Resources), Social Sciences Citation Index (Thomson Reuters Web of Science), ASSIA (CSA), IBSS (EBSCO), Sociological Abstracts (CSA), Scopus (Elsevier) and the NHS Economic Evaluation Database (NHS CRD). A trained information scientist will be used to develop and implement the searches. An example of the search strategy is in Additional file 1. In order to ensure adequate sensitivity of the search strategy, HJM piloted the search in MEDLINE (searched 8 May 2014). This resulted in 3,321 hits, of which all 9 key indicator papers [37-45] that we had identified from our knowledge prior to running the search were included.

All databases will be searched from inception to the present day. We will not exclude papers on the basis of language, country of origin or publication date. We plan to supplement the electronic database searches with website (Google) and grey literature searches (OpenGrey, Social Care Online, Prevention Information & Evidence eLibrary and Nexis UK), and we will search trial registers (http://www.controlled-trials.com) and websites of funding organisations (such as Royal Pharmaceutical Society, Pharmacy Research UK) for ongoing studies. We will hand search the bibliographies of all included studies and request relevant information on unpublished and in-progress research from key experts in the field. We will also contact study authors for unpublished data on health inequalities.

### Study selection and screening

Two reviewers will independently screen each hit from the ten searched databases against our inclusion criteria and at each stage (title/abstract screen; full paper screen) will err on the side of inclusivity. The initial screening of titles and abstracts will be conducted independently by two reviewers (HJM and SS). Included studies from both sets will be checked for agreement, and any disagreements will be resolved via discussion; if agreement cannot be reached, then the decision will be referred to a third reviewer (COM). Full-paper study inclusion will be conducted by two reviewers (from HJM, SS and COM). As before, included studies from both sets will be checked for agreement and any disagreements will be resolved via discussion; if agreement cannot be reached, then the decision will be referred to another member of the review team (AT or CS).

### Data extraction and quality appraisal

Data extraction will be conducted independently for each study by two reviewers (from AH, AT, CB, COM, CS, FFS, HJM, LN, LS and SS) using established, electronic (to ensure consistency in data extraction between reviewers) data extraction forms adapted and refined for the purposes of this review (see Additional file 2 for the draft data extraction form). Any discrepancies will be resolved through discussion between the two reviewers, and if consensus is not reached, then the decision will be referred to a third member of the review team. Before data extraction commences, all included studies will be checked using author names, study names, sample sizes and types of outcome data and those papers that report from the same study will be extracted at the same time to prevent double counting.

Data to be extracted will include details of the project (aims, settings targeted, intervention description, resources, date (started and completed), study sponsor or funding source, etc.), study details (study design, population targeted, demographics, recruitment and follow-up rates, etc.), measured outcomes and methods used, and results. Data on the organisation, implementation and delivery of interventions will also be extracted using existing methodological tools which assess the implementation of complex public health interventions [36] adapted and refined for the purposes of this review. Examples of the implementation components that will be examined include theoretical underpinning, implementation context, experience level of the intervention team (planners and implementers), consultation and/or collaboration processes (planning and delivery stages) and resources (for example, time, money, staff and equipment). We will also extract information on intervention costs and potential cost saving through intervention effects. The data extraction forms have been designed based upon a data extraction form used in our previous National Institute for Health Research (NIHR)-funded inequalities in obesity reviews [46,47]. We will pilot the new form using a sample of the studies that are to be reviewed. If major changes to the data extraction form are required, then a second phase of pilot testing will be carried out.

The methodological quality of the included studies will also be appraised independently by two reviewers using the Effective Public Health Practice Project Quality Assessment Tool for Quantitative Studies [48] as recommended by the Cochrane Public Health Review Group [49]. As before, any discrepancies will be resolved through discussion, and if consensus cannot be reached, the studies will be referred to a third reviewer. We will use the quality appraisal criteria for descriptive purposes and to highlight variations between studies in the narrative overview. Studies will be classified as ‘high’ , ‘moderate’ or ‘weak’ quality. If we are able to conduct meta-analyses, we will use ‘quality’ in the sensitivity analysis.

### Analysis and synthesis

Meta-analysis will be conducted if it is deemed appropriate to do so [30]. A meta-analysis will be considered if we identify at least three RCTs that compare the effects of a similar intervention on the same behaviour outcome. Taking smoking cessation as an example, we would consider conducting a meta-analysis where we identified at least three RCTs where the intervention was advice on smoking cessation or where advice on smoking cessation plus nicotine replacement therapy was the intervention, but we would not consider combining these two different intervention types together in a meta-analysis. In terms of behavioural outcomes, and taking weight management as an example, we would consider conducting a meta-analysis where at least three RCTs had measured dietary intake, even though different methods had been used to assess dietary intake across the studies, but we would not consider combining dietary intake and physical activity behaviour outcomes.

Stata will be used for meta-analyses using random effects model (currently version 12), and WinBUGS will be used to incorporate informative priors or external information. If meta-analyses are not possible, the synthesis will be restricted to a narrative overview of individual studies looking at the same question. We propose to construct a narrative overview using a similar format to that which we used for reporting the results of a systematic review (also funded by NIHR) on the effectiveness of individual, community and societal level interventions at reducing socio-economic inequalities in obesity [46,47]. Structured overviews of each study (in terms of population, intervention, context, outcome and study design) will be included. Forest plots within a narrative review will mainly serve as a graphical presentation of effect sizes from the studies. Meta-regression will be conducted using a random effects model, and sub-group analysis of gender and age will only be carried out if they show differential effects in a meta-regression or moderator analysis. We will also investigate publication bias by using funnel plots and Egger's test [50]. If we are able to conduct meta-analyses, we will conduct sensitivity analysis adjusted for moderators, including ‘quality’. We will assess whether any original heterogeneity (*Q*-statistic) between studies is explained by the moderators.

Socio-economic, gender and age inequalities in the effects of interventions are a secondary outcome for this review. As such, and following our previous NIHR-funded systematic reviews of the effects of interventions on health inequalities [46,47], studies included under the primary outcome that also examine differential effects of interventions with regard to individual or area-level measures of socio-economic status (education, income, occupation, social class, deprivation, poverty) or in which the intervention is targeted specifically at disadvantaged groups (living in areas of high deprivation, unemployed, low SES, low income) will be included in the health inequalities analysis. Such measures will thereby capture interventions that focus on reducing health gaps (between the most and the least affluent), shifting health gradients (the health across the whole social hierarchy) or improving the health of disadvantaged groups [51]. Similarly, we will conduct sub-group analysis by gender and age using meta-regression where possible.

Contextual data on the organisation, implementation and delivery of interventions will be extracted using existing methodological tools which assess the implementation of complex public health interventions [36], after being adapted and refined for the purposes of this review. Examples of the implementation components that will be examined include theoretical underpinning of the intervention, implementation context, experience of the intervention team (planners and implementers), consultation/collaboration processes (planning and delivery stages) and resources (e.g. time, money, staff, equipment). This information will help users of the review in translating the findings to their own policy or practice context. Our analysis will emphasise explaining heterogeneity of effects, including a moderator analyses for population features such as socio-economic status and ethnicity. If there is sufficient data, we will also take features of the interventions delivered into consideration, as intervention content might be highly heterogeneous.

## Discussion

The review aims to summarise the evidence base on the effectiveness of community pharmacy interventions on health and health behaviours in relation to weight management, smoking cessation and alcohol misuse. It will also explore if, and how, socio-economic status, gender, ethnicity and age moderate the effect of the interventions and will describe how the interventions included in the review have been organised, implemented and delivered, since context is an important factor governing the success of public health interventions. The findings from this review will have an impact on the commissioning of public health services in the UK which aim to promote healthy weight and smoking cessation and prevent excessive alcohol consumption.

## Competing interests

The authors declare that they have no competing interests.

## Authors’ contributions

AT, AKH and CB led on the conception of the study idea. AT, AKH, CB, AK and FFS participated in the study design and the development of methods. HJM assisted in the study design and participated in the development of methods, developed the search strategy and drafted the manuscript with AT. LS assisted with the development of the search strategy methods. CS and AT led on the study design and the development of methods. CS led on the management of this systematic review. All authors provided critical comments on the manuscript and contributed to the write-up. All authors have approved the final version.

## Supplementary Material

Additional file 1 This file contains details of the Medline search strategy that was executed through the Ovid platform. Click here for file

Additional file 2 This file shows the draft data extraction form that we plan to use in the systematic review process. Click here for file
