# Supplementary material for: Community pharmacy interventions for public health priorities: protocol for a systematic review of community pharmacy-delivered smoking, alcohol and weight management interventions
Source: Syst Rev. 2014 Aug 22;3:93. doi: 10.1186/2046-4053-3-93 (PMC4145162; doi:10.1186/2046-4053-3-93)
Supplement: Additional file 1 — This file contains details of the Medline search strategy that was executed through the Ovid platform. [file 2046-4053-3-93-S1.doc]

**Medline (Ovid) Search Strategy**

| **#** | **search term** |
| --- | --- |
| 1 | exp Community Pharmacy Services/ |
| 2 | Pharmacies/ |
| 3 | exp Pharmacists/ |
| 4 | exp Pharmacists' Aides/ |
| 5 | Pharmacy/ |
| 6 | chemist.tw. |
| 7 | (communit$ adj7 pharmac$).tw. |
| 8 | (office$ adj7 pharmacy$).tw. |
| 9 | ((pharmacy or pharmacist? or pharmacies) adj3 (community or counsel$ or advice or care)).tw. |
| 10 | (pharmacist? adj3 (front line or 'one to one' or face to face)).tw. |
| 11 | (pharmacist? or pharmacy or pharmacies).tw. |
| 12 | ((pharmacist? or pharmacy) adj3 (aide or aides or assistant? or staff)).tw. |
| 13 | (Pharmacist? adj2 (care or delivered)).tw. |
| 14 | (pharmacist? adj3 (counsel$ or (patient? adj2 education$) or led or intervention? or public health or diagnos$)).tw. |
| 15 | or/1-14 |
| 16 | exp Obesity/ |
| 17 | exp Body Weight/ |
| 18 | exp Body Weight Changes/ |
| 19 | exp Weight Gain/ or exp Weight Loss/ |
| 20 | (obese or obesity).tw. |
| 21 | overweight.tw. |
| 22 | weight.tw. |
| 23 | diet$.tw. |
| 24 | nutrition$.tw. |
| 25 | (physical$ adj activ$).tw. |
| 26 | exercise$.tw. |
| 27 | lifestyle$.tw. |
| 28 | (bmi$ or (body adj mass ind$)).tw. |
| 29 | (waist adj6 circumference$).tw. |
| 30 | ((weight adj2 (control or reduction) adj2 (advice or counsel$ or program$ or intervention?)) or (weight adj manag$)).tw. |
| 31 | ((overweight or obese or obesity) adj4 (Advice or counsel$ or intervention? or program$)).tw. |
| 32 | or/16-31 |
| 33 | exp Smoking/ or exp Smoking Cessation/ |
| 34 | nicotine.tw. |
| 35 | cigarette$.tw. |
| 36 | (nicotine replacement therapy or NRT).tw. |
| 37 | smoking cessation.tw. |
| 38 | smok$.tw. |
| 39 | exp "Tobacco Use Cessation"/ |
| 40 | exp Smoking Cessation/ |
| 41 | (smoking cessation or (quit$ adj2 smok$)).tw. |
| 42 | ((reduce or reducing) adj3 ('tobacco use' or cigarette? or smoking or addiction)).tw. |
| 43 | or/33-42 |
| 44 | alcohol.mp. |
| 45 | exp Alcohols/ |
| 46 | exp Alcohol Drinking/ |
| 47 | exp Alcoholism/ |
| 48 | exp Drinking Behavior/ |
| 49 | (drink$).tw. |
| 50 | beer.tw. |
| 51 | wine.tw. |
| 52 | ethanol.tw. |
| 53 | drunk.tw. |
| 54 | (addict$ or (alcohol adj2 (abus$ or misus$))).tw. |
| 55 | alcohol$.tw. |
| 56 | drunk$.tw. |
| 57 | intoxicat$.tw. |
| 58 | or/44-57 |
| 59 | 32 or 43 or 58 |
| 60 | (animals not humans).mp. |
| 61 | 59 not 60 |
| 62 | 15 and 60 |
| 63 | limit 62 to humans |
