# Supplementary figures and images for: Community pharmacy interventions for public health priorities: protocol for a systematic review of community pharmacy-delivered smoking, alcohol and weight management interventions
Source: Syst Rev. 2014 Aug 22;3:93. doi: 10.1186/2046-4053-3-93 (PMC4145162; doi:10.1186/2046-4053-3-93)

**Additional File 2: draft data extraction form**


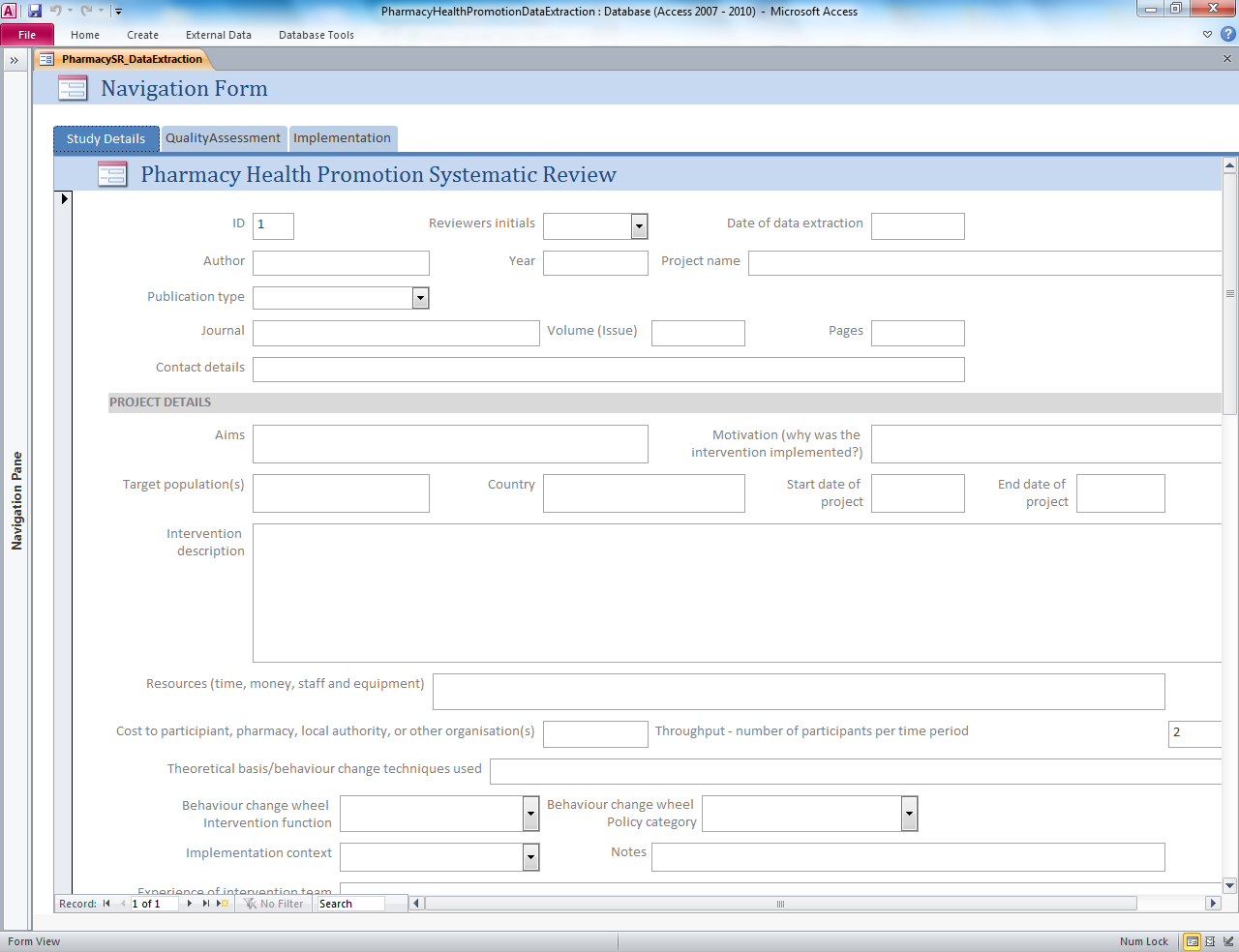

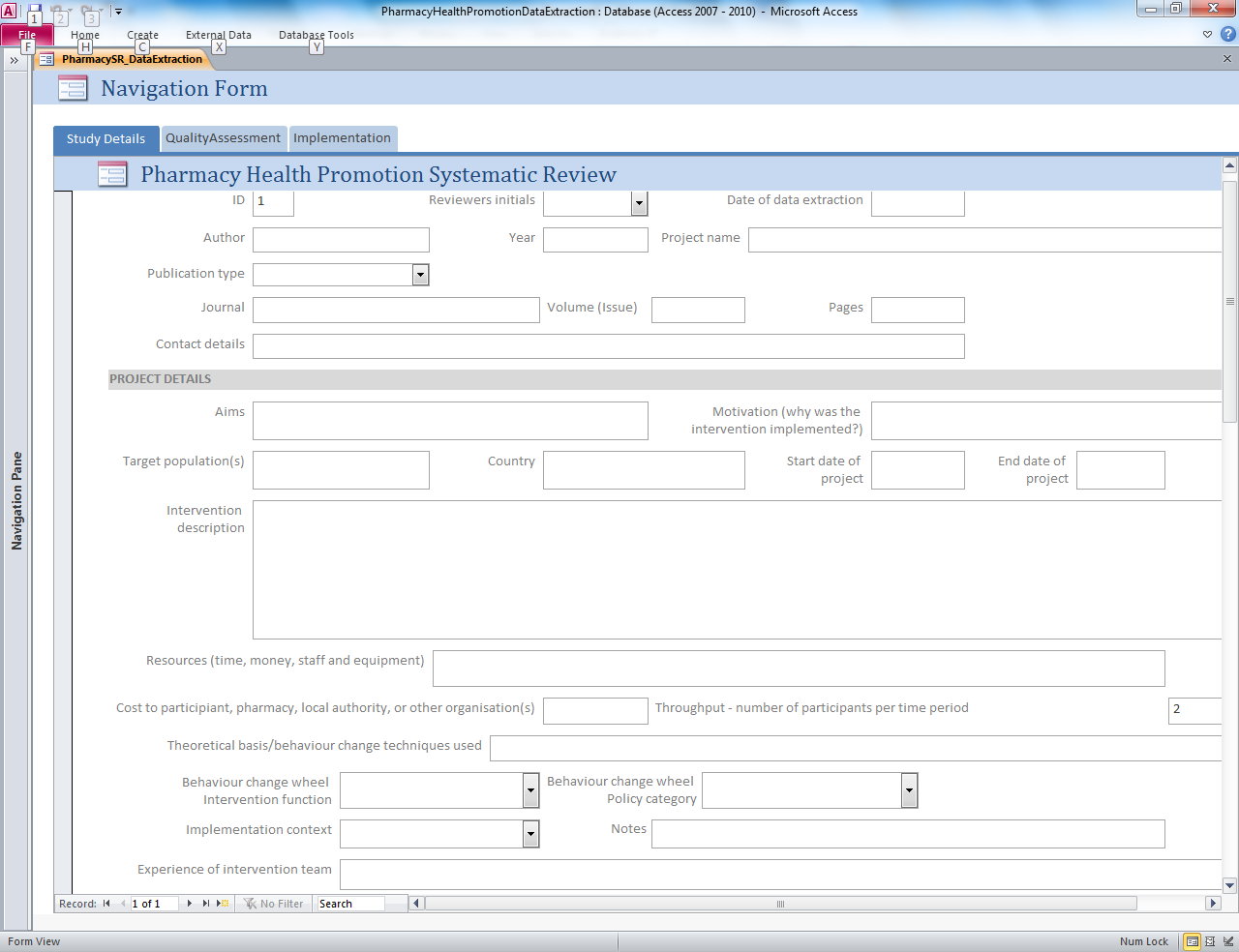

Supplement: Additional file 2 — This file shows the draft data extraction form that we plan to use in the systematic review process. [file 2046-4053-3-93-S2.docx]
